# Evans Blue Inhibits HBV Replication Through a Dual Antiviral Mechanism by Targeting Virus Binding and Capsid Assembly

**DOI:** 10.3389/fmicb.2019.02638

**Published:** 2019-11-14

**Authors:** Yu Xiao, Chunlan Liu, Wei Tang, Haiwei Zhang, Xulin Chen

**Affiliations:** ^1^State Key Laboratory of Virology, Wuhan Institute of Virology, Chinese Academy of Sciences, Wuhan, China; ^2^University of Chinese Academy of Sciences, Beijing, China; ^3^Guangdong Key Laboratory of Virology, Institute of Medical Microbiology, Jinan University, Guangzhou, China

**Keywords:** HBV, inhibitor, Evans blue, binding, capsid assembly

## Abstract

Chronic hepatitis B (CHB) is a global health problem caused by human hepatitis B virus (HBV). Current treatment with interferons and nucleos(t)ide analogs (NAs) can cause population tolerance and drug resistance. Therefore, new antiviral drugs, especially those targeting host factors, are urgently needed. Here, we identified Evans blue as a new HBV inhibitor by screening an FDA drug library using Huh7D^hNTCP^ cells and confirmed the antiviral activity in primary human hepatocytes and human sodium taurocholate cotransporting polypeptide (hNTCP)-transfected porcine primary hepatocytes. Our efficacy study showed that Evans blue has an IC_50_ of 2 μM against HBV infection in Huh7D^hNTCP^ cells, and no apparent toxicity at up to 1000 μM. The IC_50_ of Evans blue against HBV in primary human hepatocytes was approximately 5 μM. Mechanism studies revealed that Evans blue has a dual anti-HBV effect. It inhibits both the binding of viral preS1 to host cells through the host factor NTCP and the virus capsid assembly by targeting the host factor BK channel. The K_D_ of the direct interaction between Evans blue and NTCP is 8.82E-8 M. Evans blue can suppress capsid assembly at micromolar concentrations by reducing the cytosolic calcium ion concentration. Since the antiviral effects on HBV binding and assembly are both achieved through targeting host factors, Evans blue inhibits the infection of nucleos(t)ide analog drug-resistant HBV strains in Huh7D^hNTCP^ cells. Taken together, our results suggest that Evans blue may be a promising anti-HBV drug candidate in the classes of both entry and assembly inhibitors.

## Introduction

Hepatitis B virus (HBV) infection is a global health threat that is estimated to cause chronic hepatitis and hepatocellular carcinoma (HCC) in over 240 million people worldwide (Ott et al., 2012). Despite the reduction in carriers of the hepatitis B surface antigen (HBsAg) due to widespread vaccination (Seeger and Mason, 2000), many patients with chronic hepatitis B (CHB) remain.

Hepatitis B virus studies are limited by a lack of reliable *in vitro* infection cell models, including liver cells, many of which cannot support HBV infection due to a lack of HBV specific receptors or co-receptors (Glebe and Urban, 2007). Therefore, the identification of HBV receptors is critical. Several cellular proteins have been suspected but not confirmed as HBV receptor molecules. Sodium taurocholate cotransporting polypeptide (NTCP), a hepatic membrane transporter involved in bile acid uptake, has been reported as an HBV receptor. Exogenous NTCP expression rendered non-susceptible hepatocarcinoma cells susceptible to HBV infections (Yan et al., 2012).

Hepatitis B virus is a small enveloped DNA virus belonging to the family of hepadnaviridae that is highly species-specific, infecting only human and some non-human primate hepatocytes. During the life cycle of HBV, highly sulfated proteoglycans (HSPGs) play an essential role in virus recognization (Schulze et al., 2007; Leistner et al., 2008). HBV first binds to HSPGs and then to NTCP. After endocytosis and fusion with cells, the viral nucleocapsid containing relaxed circular partially double-stranded DNA (rcDNA) with covalently linked polymerase is released and the nucleocapsid is transported on microtubules to nuclear pore complex adaptor proteins. Then, the rcDNA is released into the nucleoplasm and repaired to form covalently closed circular DNA (cccDNA) with the help of host specific factors. All viral RNAs necessary for protein production and viral replication are transcribed from the cccDNA, and the pregenomic RNA (pgRNA) recruits the core protein and polymerase via its epsilon stem-loop structure to assemble an RNA-containing nucleocapsid. During nucleocapsid maturation, rcDNA is transcribed from the pgRNA. The DNA-containing nucleocapsids can then either be re-imported into the nucleus to form additional cccDNA molecules or enveloped for secretion (Urban et al., 2010).

Since the entire life cycle of HBV and the mechanisms of HBV-induced HCC are not clearly understood (Dandri and Petersen, 2016; Levrero and Zucman-Rossi, 2016), treatments for HBV infections are limited. Interferon-α, nucleos(t)ide analogs (NAs) are commonly used to treat hepatitis B (Grimm et al., 2011), but these agents cannot completely eliminate HBV from infected cells (Tenney et al., 2004). Preventing cccDNA formation and eliminating established cccDNA are the major challenges in anti-HBV treatment (Levrero et al., 2009). Although Interferon-α and lymphotoxin-β receptors mediated upregulation of APOBEC3A and APOBEC3B cytidine deaminases can induce cccDNA degradation, the efficiency is poor (Lucifora et al., 2014). In a previous attempt to identify cccDNA inhibitors, only CCC-0975 and three hydrolyzable tannins (punicalagin, punicalin, and geraniin) were found to inhibit cccDNA formation (Cai et al., 2012; Liu et al., 2016). In sum, current HBV therapies are very limited, so new targets and drugs are urgently needed for anti-HBV therapy.

NTCP is a sodium-dependent bile salts transporter that has recently been confirmed as an HBV/HDV receptor. Hepatocytes take up bile salts from the portal blood in a sodium-dependent manner through NTCP. The functions of NTCP in sodium taurocholate transportation, myr-preS1 peptide binding, and HBV infection show comparable kinetics (Konig et al., 2014). However, the expression of NTCP is very low in the currently available hepatocyte cell lines, such as HepG2 and Huh-7, compared to primary human hepatocytes. Expressing NTCP in these cell lines makes it possible to study the HBV life cycle from entry to the secretion of progeny virus (Tsukuda et al., 2015). Since NTCP was identified as one of HBV’s entry receptors, several compounds targeting NTCP have been reported to inhibit HBV entry. Among these, cyclosporin A (CsA) (Nkongolo et al., 2014; Shimura et al., 2017) and vanitaracin A (Kaneko et al., 2015) can directly interact with NTCP to interfere with HBV binding. Also, EGCG (Huang et al., 2014), a flavonoid present in green tea extract belonging to the subclass of catechins, and Ro41-5253 (Tsukuda et al., 2015) can inhibit HBV entry by degrading NTCP. However, these compounds are not suitable for further development as new anti-HBV drugs due to their insolubility and toxicity *in vivo*.

In the present study, we screened an FDA approved drug library consisting of 1280 compounds for anti-HBV activity and identified Evans blue as a potent HBV inhibitor. Evans blue can inhibit 50% of HBV infection at a concentration of 2 μM on Huh7D^hNTCP^ cells, and the CC_50_ is higher than 1000 μM. Evans blue can also inhibit HBV infection of primary human hepatocytes (PHH) and primary porcine hepatocytes transfected with human NTCP. Our mechanism of action study indicates that Evans blue has a dual antiviral effect on HBV preS1 binding to NTCP by interacting with NTCP directly and HBV capsid assembly by reducing cytosolic Ca^2+^ levels. Considering its safety for *in vivo* use, favorable solubility, and dual antiviral effect targeting host factors which is linked to a much higher drug-resistant barrier, Evans blue may be a promising anti-HBV drug candidate in the classes of entry inhibitors and assembly inhibitors.

## Materials and Methods

### Drugs and Antibodies

Entecavir was purchased from Melone Pharma, Corp (Dalian, China). Dexamethasone, hydrocortisone, EGF recombinant human protein, DMSO, G418, DOX, thapsigargin, and the anti-SLC10A1 antibody were obtained from Sigma (St. Louis, MO, United States). Evans blue was purchased from Aladdin (China), PEG8000 was purchased from Amresco (Solon, OH, United States). Direct blue 1, Direct blue 2, Direct blue 6, Direct blue 14, and Direct blue 71 were purchased from Acmec Biochemical (Shanghai, China). Direct blue 15 and Direct blue 218 were purchased from Toronto Research Chemicals (Canada). PNGaseF was obtained from New England Biolabs (Ipswich, MA, United States). The anti-HBc antibody was purchased from DAKO (Carpinteria, CA, United States). His-tag, GAPDH, and β-actin antibodies were purchased from Beyotime (Shanghai, China). The purities of the drugs were all > 98%.

### Cell Lines and Cell Culture

The HepAD38 cell line (Ladner et al., 1997) was a generous gift from Christoph Seeger (Fox Chase Cancer Center, Philadelphia, PA, United States). The Huh7D^hNTCP^ cell line was a gift from Xinwen Chen (Wuhan Institute of Virology, CAS, China). The PHH were purchased from the Research Institute for Liver Diseases (Shanghai) Co., Ltd. The primary porcine hepatocytes (PPH) and LV-hNTCP001 were purchased from Liver-Biotechnology (Shenzhen) Co., Ltd. Rat type I collagen was purchased from Beijing East Mo Biotechnology, Co., Ltd. HepAD38 cells were maintained in Dulbecco’s modified Eagle’s medium/F-12 (DMEM/F12; Gibco, 10% FBS; Gibco, 100 U/mL penicillin, 100 U/mL streptomycin, 200 μg/mL G418 and 1 μg/mL DOX) to suppress HBV replication. Before the experiments, the cells were cultured in DMEM/F12 without DOX for 3 days. Huh7D^hNTCP^ cells were cultured as previously described (Zhou et al., 2017). PHH cells were cultured in InVitroGRO HI Medium (BioreclamationIVT). The cell culture of PPH, viral transduction, and HBV infection were conducted as previously described (Lempp et al., 2017).

### HBV Preparation and Infection

HepAD38 cells were used to produce HBV particles (genotype D ayw serotype). The cells were cultured in DMEM/F12 without DOX for 4 days, and then the media were replaced with HBV-producing medium (William’s E Medium, Gibco, 5%FBS, 100 U/mL penicillin, 100 U/mL streptomycin, 2% DMSO). Cell supernatants were collected every 4 days and precipitated with 8% PEG8000 for 8–12 h at 4°C, followed by centrifugation for 1 h (4°C, 8000 rpm). The virus-containing pellets were resuspended with William’s E medium (1XITS, Gibco, 18 ng/mL hydrocortisone, 40 ng/mL dexamethasone, 10 ng/mL EGF, 100 U/mL penicillin, 100 U/mL streptomycin, 2% DMSO), and HBV genome copies were determined by real-time PCR. Huh7D^hNTCP^ cells were infected with HBV at > 1000 genome equivalents (GEq)/cell. PHH and PPH were infected with HBV at > 500 GEq/cell. After being an incubation with HBV, the cell plates were centrifuged for 1 h (4°C, 1000 *g*) (Yan et al., 2015) to enhance infection.

### Extraction of HBV Total DNA, cccDNA, and Capsid DNA

Cells were lysed with lysate buffer containing 10 mM Tris-HCl (pH 7.5), 150 mM NaCl, 10 mM EDTA and 0.7% SDS. After 30 min incubation at room temperature, samples were divided into two equivalents, one for total DNA preparation and the other for cccDNA preparation (Werle-Lapostolle et al., 2004). For the preparation of total DNA, the lysate was treated with 0.4 mg/mL proteinase K for 8 h at 58°C and then extracted with phenol/chloroform twice. The total DNA in the supernatant was precipitated with ethanol overnight at −20°C and dissolved in TAE. For the preparation of cccDNA, the lysate was treated with 0.25 volume of 5M NaCl at 4°C overnight. The supernatant was collected by centrifugation at 13000 rpm for 30 min at 4°C. After two extractions with phenol/chloroform, the supernatant was precipitated with ethanol overnight at −20°C and the cccDNA was dissolved in double-distilled water and treated with PSAD (plasmid-safe ATP-dependent DNase) to eliminate rcDNA, dsDNA, and ssDNA. For the preparation of capsid DNA, cells were lysed at room temperature for 30 min with lysate buffer containing 10 mM Tris-HCl (pH 7.5), 1 mM EDTA, 100 mM NaCl and 0.1% NP40, and then treated with 100 U/mL DNase I overnight at 37°C. Finally, buffer containing 10 mM EDTA, 0.5% SDS, and 0.4 mg/mL Proteinase K was added to lyse the capsid, followed by DNA extraction using the same protocol for total DNA preparation.

### Primers for Real-Time PCR

DNA copy number was determined by real-time PCR analysis. Primers for quantification of HBV total DNA were 5′-ACTCACCAACCTCTTGTCCT-3′ and 5′-GACAAACGGGCAACATACCT-3′; the primers for internal control GADPH were 5′-GAAGGTGAAGGTCGGAGTC-3′ and 5′-GAAGATGGTGATGGGATTTC-3′ (Zhang et al., 2016). The selective primers for cccDNA were 5′-CTCCCCGTCTGTGCCTTCT-3′ and 5′-GCCCCAAAGCCACCCAAG-3′, the internal control primers for mitochondrial DNA were 5′-CCCCACAAACCCCATTACTAAACCCA-3′ and 5′-TTTCATCATGCGGAGATGTTGGATGG-3′ (Liu et al., 2016).

### Detection of HBeAg and HBsAg

The levels of HBsAg and HBeAg in the supernatants of cell cultures were determined using ELISA Kits (Kehua Bio-Engineering, Corp.) according to the manufacturer’s recommendations.

### Cytotoxicity Assay

Cells were seeded in 96-well plates at a density of 1 × 10^4^ cells per well and grew to 80% confluence 24 h after seeding. A concentration gradient of Evans blue was added and the cells were cultured for an additional 72 h. The supernatants were removed and the cells were washed three times with PBS. Cells in each well were then incubated with 100 μL of diluted Alamar blue reagent at 37°C for 1 h. The fluorescence was measured at an excitation wavelength of 530–560 nm and an emission wavelength of 590 nm using a Perkin Elmer Envision Multi-label Plate Reader.

### Western Blot Assay and Viral Particle Gel Assay

Western blot analysis and HBV particle gel assay were performed as described previously (Zhang et al., 2016). For detection of NTCP, samples were treated with PNGaseF according to the manufacturer’s recommendations, followed by SDS-PAGE and then transferred to a membrane; after a blockage, the membrane was blotted with anti-SLC10A1 antibody and HRP-tagged secondary antibody subsequently. For HBV capsid detection, cells in 6 well plates were lysed with 300 μL lysate buffer [10 mM Tris-HCl (pH 7.6), 0.1% NP-40, 100 mM NaCl, and 1 mM EDTA] at room temperature for 30 min and cell debris was removed by centrifugation at 5000 *g* for 10 min. The cell lysate was fractionated by 1.5% native agarose gel electrophoresis at 70 V for 3 h in TAE buffer and then transferred to a Nylon membrane using the capillary transfer method with SSC buffer overnight. Capsid detection was followed by western blotting using anti-HBcAg antibody and SuperSignal West Femto Maximum Sensitivity Substrate (Thermo).

### PreS1 Binding Assay

Cells were incubated with FITC-preS1-myr (type C), preS1-myr (type C) (Chinapeptides, Co., Ltd.) and Evans blue at 37°C for 30 min. Three washes with PBS removed free FITC-preS1-myr and Evans blue. Then the cells were fixed with 4% paraformaldehyde and stained with DAPI (Tsukuda et al., 2015). The FITC and DAPI signals were detected using a high-content analysis system Operetta (PerkinElmer).

### Expression and Purification of Recombinant Human-NTCP and NTCP-Drug Interaction Assay

For the preparation of recombinant NTCP, pEnter-hNTCP was transfected into HEK293T cells. Cells were collected 72 h post-transfection and NTCP was purified by following a His-Tag purification protocol. The concentration of NTCP was determined by BCA assay. NTCP was dialyzed against the assay buffer (PBS containing 0.1% BSA and 0.02% TW20) at 4°C for 24 h, then incubated with biotin-LCLC-NHS (Thermo Fisher) at a molar ratio of 1:5 at room temperature for 3 h. Free biotin-LCLC-NHS was removed by dialysis. The interaction between NTCP and Evans blue was detected on an Octet RED system (ForteBio, United States). PreS1 was used as a positive control. BSA was used as a control protein labeled with biotin-LCLC-NHS.

### HBV Binding, Endocytosis/Fusion, and Virucidal Assays

In the HBV binding assay, Huh7D^hNTCP^ and hNTCP-PPH cells were incubated with Geq = 2000 HBVs together with Evans blue at 4°C for 2 h. Unbound virus and compounds were washed off with PBS for three times and the cells were cultured for an additional 5 days. HBV infectivity was determined by supernatant HBeAg level and HBV total DNA.

In the HBV endocytosis/fusion assay, Huh7D^hNTCP^ and hNTCP-PPH cells were incubated with Geq = 2000 HBVs at 4°C for 2 h. Free HBVs were washed with PBS for three times, and Evans blue was then added, followed by incubation at 37°C for 2 h. The cells were maintained for 5 days after three washes with PBS removed the compounds. HBV infectivity was determined by supernatant HBeAg levels and HBV total DNA levels.

In the virucidal assay, HBVs were incubated with Evans blue at room temperature for 2 h, and then Evans blue was removed by centrifugation using an Amicon Ultra-15 centrifuge (Millipore UFC910096). Huh7D^hNTCP^ or hNTCP-PPH cells were infected with the HBV at Geq = 2000, and HBV infectivity was determined by HBeAg level and HBV total DNA level at 5 dpi.

### Measurement of Cytosolic Calcium

Briefly, HepAD38 cells were seeded in 96-well plates at 2 × 10^4^ cells per well and cultured in DMEM/F12 for 24 h. Cells were then washed with HBSS buffer three times and incubated with 5 μM Fluo4-AM containing 0.05% PluronicF-127 (Yeasen Biotech, Co., Ltd.) at 37°C for 1 h. The cells were washed three times with HBSS and incubated at 37°C for another 30 min to make the fluorescence probe completely de-esterifying. The cells were then incubated with serially diluted compounds and the Ca^2+^ levels were determined based on the fluorescence (excitation wavelength 480 nm, emission wavelength 525 nm) measured using a Perkin Elmer Envision Multi-label Plate Reader.

## Results

### The Antiviral Effect of Evans Blue Against HBV Infection in Huh7D^hNTCP^ Cells

**FIGURE 1:**
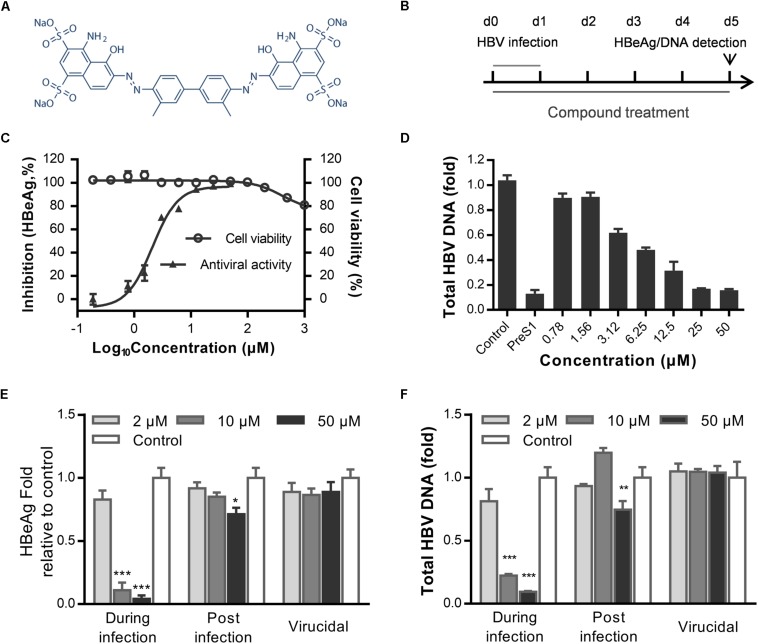
Evans blue inhibits HBV infection. **(A)** The chemical structure of Evans blue. **(B)** In the initial experimental setup, cells were infected at day 0 with an HBV inoculum of 1000 GEq/cell, which was washed off 16 h later. Then cells were maintained in the maintenance medium for another 5 days. Supernatant HBeAg level was detected by ELISA, total HBV DNA was analyzed by real-time PCR at 5 dpi. Evans blue was present during the entire experiment. **(C)** For the determination of cytotoxicity, Huh7D^hNTCP^ cells were cultured in the presence of serially diluted Evans blue for 72 h, and cell viability was measured using Alarma blue. For the determination of the antiviral effect, cells were infected with HBV and treated with serially diluted Evans blue for 5 days, and supernatant HBeAg levels were measured by ELISA. **(D)** Huh7D^hNTCP^ cells were infected and treated the same as in **(C)**, and at 5 dpi, HBV total DNAs were quantified by real-time PCR. Human GADPH was used as an internal reference. **(E,F)** Determination of the antiviral effects during infection, post-infection, and the virucidal effect. For determining antiviral effect during infection, cells were treated with 2, 10, and 50 μM of Evans blue during infection and then washed with PBS three times to remove the unbound viruses and compounds. Cells were cultured with maintaining medium, and the levels of HBeAg in the supernatant and total HBV DNA in cells were detected at 5 dpi. For determining the antiviral effect post-infection, cells were infected with HBVs for 16–20 h and then washed three times with PBS. Cells were cultured in the presence of Evans blue for 5 days. The levels of the supernatant HBeAg or total HBV DNA in cells were determined. For determining the virucidal effect, a virucidal assay was conducted as described in Section “Materials and Methods.” The supernatant HBeAg **(E)** and total DNA **(F)** levels were determined using ELISA and RT-PCR, respectively. Statistical significances were determined using two-tailed test (^∗^*P* < 0.05, ^∗∗^*P* < 0.01, ^∗∗∗^*P* < 0.001).

To identify novel HBV inhibitors, we screened a US drug library (MicroSource, Gaylordsville, CT, United States) of 1280 compounds that had mostly been approved as non-hepatitis B therapies. We had previously identified antiviral compounds against the HBV using HepAD38 cells (Zhang et al., 2016). Herein, we identified Evans blue (Figure 1A) as a new HBV inhibitor using the Huh7D^hNTCP^ cell line, which supports the entire life cycle of HBV. As shown in Figure 1B, Huh7D^hNTCP^ cells were incubated with HBV, and the cell culture supernatants were collected for HBeAg detection by ELISA, and the HBV DNA was extracted and measured by real-time PCR. Due to the limitations of the cell line, we did not detect HBsAg levels (Zhou et al., 2017). We found that micromolar concentrations of Evans blue dramatically reduced the levels of HBeAg (Figure 1C) and HBV DNA (Figure 1D). The IC_50_ was 2.1 and 6.25 μM on HBeAg and HBV total DNA, respectively. No apparent toxicity was observed for Evans blue at up to 1000 μM. These results indicate that Evans blue is a highly selective HBV inhibitor with a selectivity index (SI) (CC_50_/IC_50_) of at least 500 in Huh7D^hNTCP^ cells. To explore which stage of HBV is blocked by Evans blue, we tested the effect of Evans blue on HBV infection and replication next. Huh7D^hNTCP^ cells were treated during infection, and post-infection, respectively, HBeAg and HBV DNA levels were measured 5 days post-infection (dpi). We found that Evans blue inhibited HBV infection and inhibited HBV replication (post-infection). Ten μM Evans blue could inhibit HBV infection completely when treated during infection. However, 50 μM of Evans blue caused only 30% inhibition on HBeAg level and 0.3 fold decrease in total HBV DNA when treated post-infection, suggesting that Evans blue inhibits infection more efficiently than post-infection. We assessed the potential virucidal effect of Evans blue by incubating compound with HBV before infection. The results showed that Evans blue did not disrupt HBV infectivity (Figures 1E,F). These data indicate that Evans blue inhibits HBV infection and replication in Huh7D^hNTCP^ cells.

### Evans Blue Inhibits HBV preS1 Binding to Huh7D^hNTCP^ Cells

**FIGURE 2:**
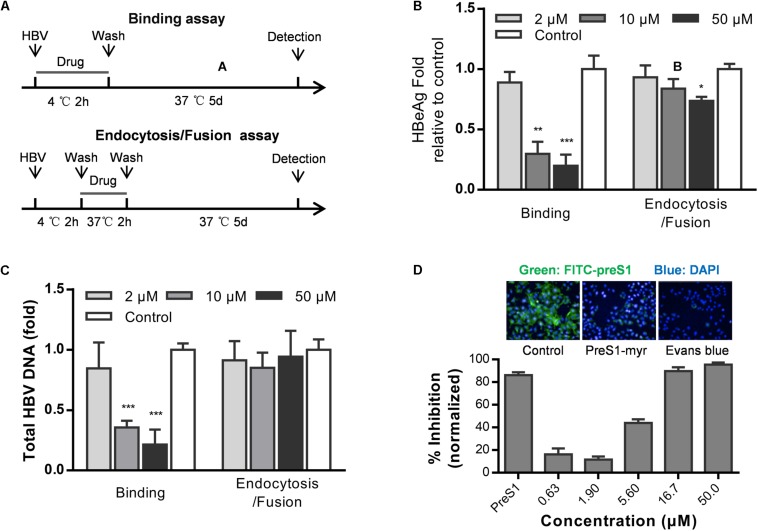
Evans blue inhibits the binding of HBV preS1 to host cells. **(A)** Experimental setup, binding assay (upper) and endocytosis assay (lower). **(B,C)** Huh7D^hNTCP^ cells were incubated with HBVs at 4°C for 2 h to enable binding but without endocytosis. Then the unbound HBVs were washed out, and cells were cultured at 37°C for 2 h to allow HBV entry. The cells were cultured for 16 h to allow HBV infection. At dpi 5, supernatant HBeAg levels and HBV total DNA levels were measured. **(D)** Cells were pretreated with or without Evans blue, the FITC-preS1 binding was detected using a PerkinElmer Operetta. preS1 at 0.1 μM was used as a positive control. The total fluorescence intensity was determined for each concentration. Statistical significance was determined using Two tails Student’s *t*-test (^∗^*P* < 0.05, ^∗∗^*P* < 0.01, ^∗∗∗^*P* < 0.001).

Hepatitis B virus infects cells by three steps: HBV attaches to heparan sulfate proteoglycans (HSPGs), binds to NTCP, and enters cells via endocytosis (Hayes et al., 2016). To examine which step in HBV entry is inhibited by Evans blue, we performed a time of addition assay as showed in Figure 2A. Evans blue was added to the medium and cells were incubated at 4°C for 2 h to allow HBV binding or at 37°C to allow the endocytosis/fusion of HBV. Results showed that Evans blue could inhibit HBV binding to cells, which is represented by the concentration-dependent reduction in both HBeAg and DNA levels. However, Evans blue inhibited very inefficiently the bound HBV from internalization into cells, suggesting that Evans blue had no apparent antiviral effect once HBV had entered the cells through endocytosis (Figures 2B,C). These data indicate that Evans blue may inhibit HBV binding to cells. Considering the function of NTCP, we used a preS1-binding assay to mimic HBV binding. Huh7D^hNTCP^ cells were incubated with both FITC-preS1 and Evans blue, nuclei were stained with DAPI, and 0.1 μM myr-preS1 was used as a positive control. We found that Evans blue inhibited the binding of FITC-preS1 to cells in a concentration-dependent manner with an IC_50_ of approximately 5 μM (Figure 2D). Evans blue completely suppressed the binding of FITC-preS1 to cells at 50 μM. Our results suggest that Evans blue can block preS1-mediated HBV binding to Huh7D^hNTCP^, and this mechanism of action may involve NTCP.

### Anti-HBV Effect of Evans Blue in Primary Human Hepatocytes and hNTCP-Transfected PPH

**FIGURE 3:**
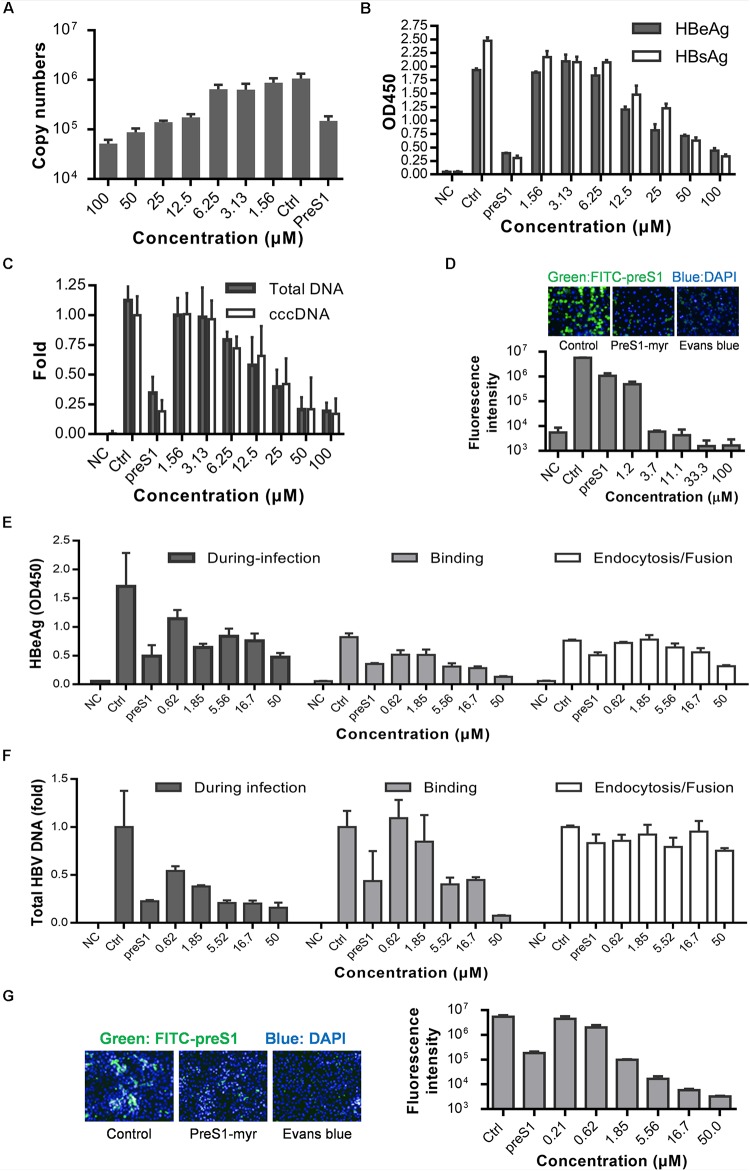
Anti-HBV effects of Evans blue on primary human hepatocytes and human NTCP transfected PPH. **(A–C)** PHH was infected by HBV particles at Geq = 1000, and cultured for 5 days in the presence of compounds. Supernatant HBeAg, HBsAg, supernatant HBV copies, total HBV DNA, and cccDNA level at 5 dpi were determined by ELISA and RT-PCR, respectively. **(D)** PHH were incubated with 0.1 μM FITC-preS1 and compounds at 37°C for 1 h, and then compounds were removed by three washes with PBS, and cells were stained with DAPI for 30 min. **(E,F)** Time of addition assay was conducted on hNTCP-PPH infected by HBV. HBeAg and total DNA were determined by ELISA and RT-PCR, respectively. **(G)** A preS1 binding assay was conducted on hNTCP-PPH in the presence or absence of Evans blue.

Although a few HBV infection cell models are available, such as the HepaRG (Gripon et al., 2002) and HepG2-NTCP-C4 cell lines (Iwamoto et al., 2014) and human hepatocytes isolated from humanized mice (Ishida et al., 2015), the gold standard cell model for HBV replication studies is still PHH model. Therefore, we next tested whether Evans blue could inhibit HBV infection of PHH. Cells were incubated with Evans blue and HBV, and the supernatant HBeAg and HBsAg were measured at 3 and 5 dpi. The total HBV DNA, progeny virus, and cccDNA levels at 5 dpi were measured by real-time PCR. As shown in Figure 3A, Evans blue could reduce supernatant HBV copy numbers dose-dependent, more than 90% of progeny HBV was eliminated at 100 μM. In Figures 3B,C, HBeAg, HBsAg, HBV total DNA, and cccDNA levels also decreased with increasing Evans blue concentrations. The IC_50_ of Evans blue for HBeAg was 12.18 and 20.99 μM for HBsAg in PHH. Real-time PCR analysis showed that 25 μM Evans blue can reduce more than half of HBV total DNA as well as cccDNA. In a FITC-preS1 binding assay on PHH, we found that the inhibitory effect on FITC-preS1 binding was greater than on Huh7D^hNTCP^ (Figures 2D, 3D). Evans blue inhibited nearly 90% of FITC-preS1 binding at as low as 1.2 μM, and the positive control, 0.1 μM preS1, exhibited a similar effect to 1.2 μM of Evans blue. These data indicate that Evans blue interferes with preS1 binding to PHH and inhibits HBV infection in PHH.

To identify whether Evans blue acts on other unknown cell factors that directly participate in HBV preS1 binding and to further evaluate the antiviral effect of Evans blue on HBV binding and infection, we used a non-human hepatocytic cell line, human NTCP transfected porcine primary hepatocytes (NTCP-PPH). As HBV is highly species-specific, and the only receptor-related with HBV on this cell line is human-NTCP, we speculated that Evans blue would not be able to inhibit HBV infection on NTCP-PPH if it does not act on NTCP. PPH were plated in collagen I coated 96-well plates and transfected with LV-hNTCP001 as described in Section “Materials and Methods.” Three days after transfection, the PPH were used for HBV infection. The time of addition experiment was conducted as shown in Figure 2A to determine the HBeAg and HBV total DNA levels (Figures 3E,F). We found that HBV infection was almost completely inhibited by the addition of Evans blue during the infection or binding stage, but was not affected by Evans blue addition during endocytosis/fusion. These results are consistent with our observations of the anti-HBV effects of Evans blue in Huh7D^hNTCP^ and PHH. It is worth noting that we found Evans blue can inhibit the binding of FITC-preS1 to the NTCP on both human hepatocyte PHH and non-human hepatocyte PPH (Figures 3D,G). Based on these findings, we hypothesized that Evans blue inhibits HBV infection by blocking the binding of the virus to NTCP.

### Evans Blue Interacts Directly With NTCP but Does Not Degrade It

**FIGURE 4:**
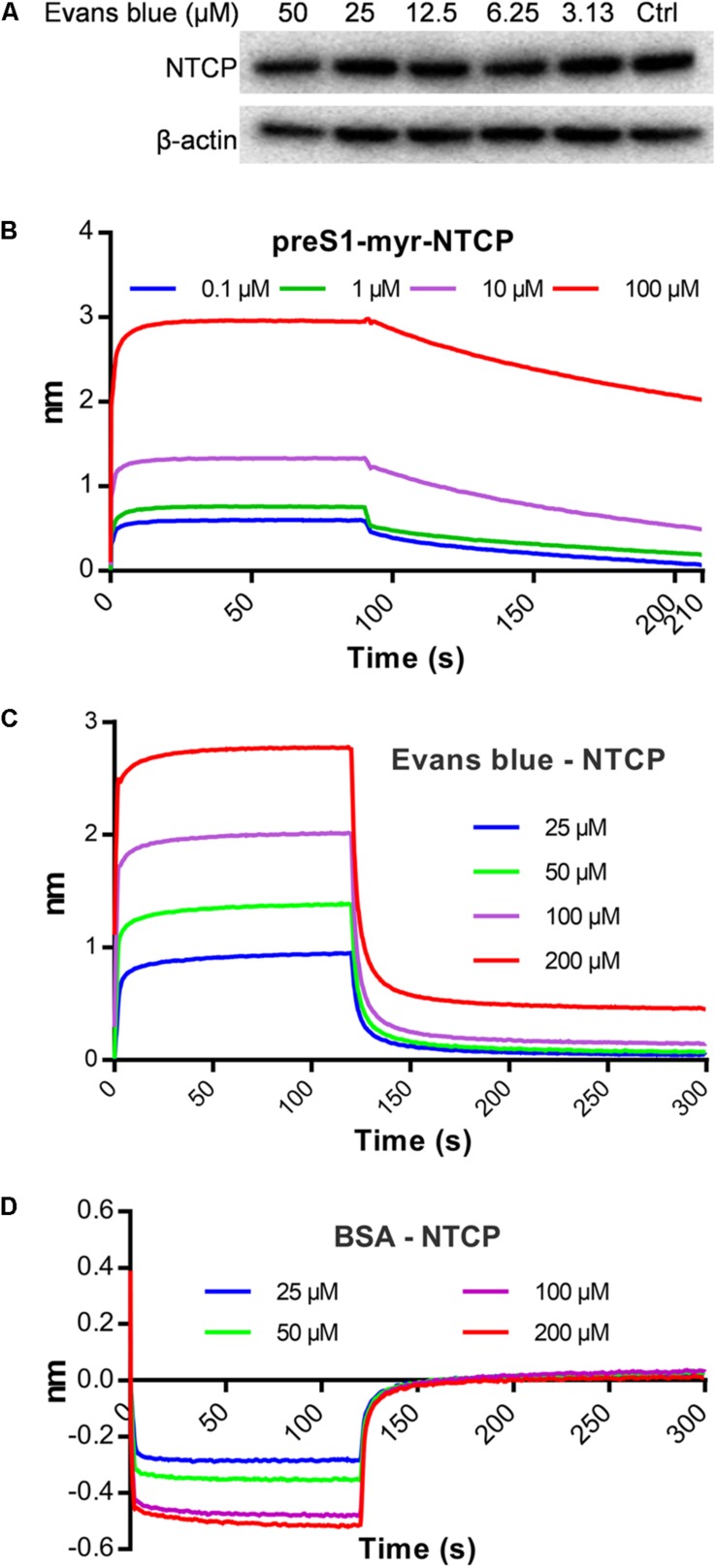
Evans blue does not degrade NTCP but directly interacts with NTCP. **(A)** Huh7D^hNTCP^ cells were treated with different concentrations of Evans blue for 72 h, cell lysates were digested with PNGaseF, and the levels of NTCP were determined by Western blot. Beta-actin was used as an internal reference marker. **(B–D)** A molecule interaction assay was conducted as described in Section “Materials and Methods.” The shift of wavelength represents the strength of the interaction between the test molecules. PreS1 **(B)** and BSA **(D)** were used as positive and negative controls, respectively. Curves correspond to the phases of association and dissociation of compounds at various concentrations on NTCP and BSA anchored to the sensor chip.

A few HBV entry inhibitors inhibit preS1 binding by interacting with NTCP (Nkongolo et al., 2014; Kaneko et al., 2015; Shimura et al., 2017) or degrading it Huang et al. (2014). To assess the influence of Evans blue on preS1 binding to NTCP, we determined the NTCP levels upon Evans blue treatment and assayed for a direct interaction of NTCP with Evans blue. Western blot results showed that NTCP was not degraded upon Evans blue treatment (Figure 4A). To study the interaction between NTCP and Evans Blue, recombinant His-tagged NTCP was purified and conjugated with biotin and used to measure the interaction with preS1 as described in Section “Materials and Methods.” PreS1 exhibited a concentration-dependent interaction with recombinant His-tagged NTCP (K_D_ = 5.56E-9 M) (Figure 4B), suggesting that the recombinant His-tagged NTCP retains its biological function of large envelope protein (LHB) binding. As expected, Evans blue did not interact with BSA even at high concentrations (K_D_ = 4.68E-4 M) (Figure 4D). However, it did interact with NTCP in a concentration-dependent manner (K_D_ = 8.82E-8 M, Figure 4C). Thus, our results clearly show that Evans blue binds NTCP, a receptor for HBV entry.

### Evans Blue Inhibits HBV Capsid Assembly by Influencing Ca^2+^ Outflow

**FIGURE 5:**
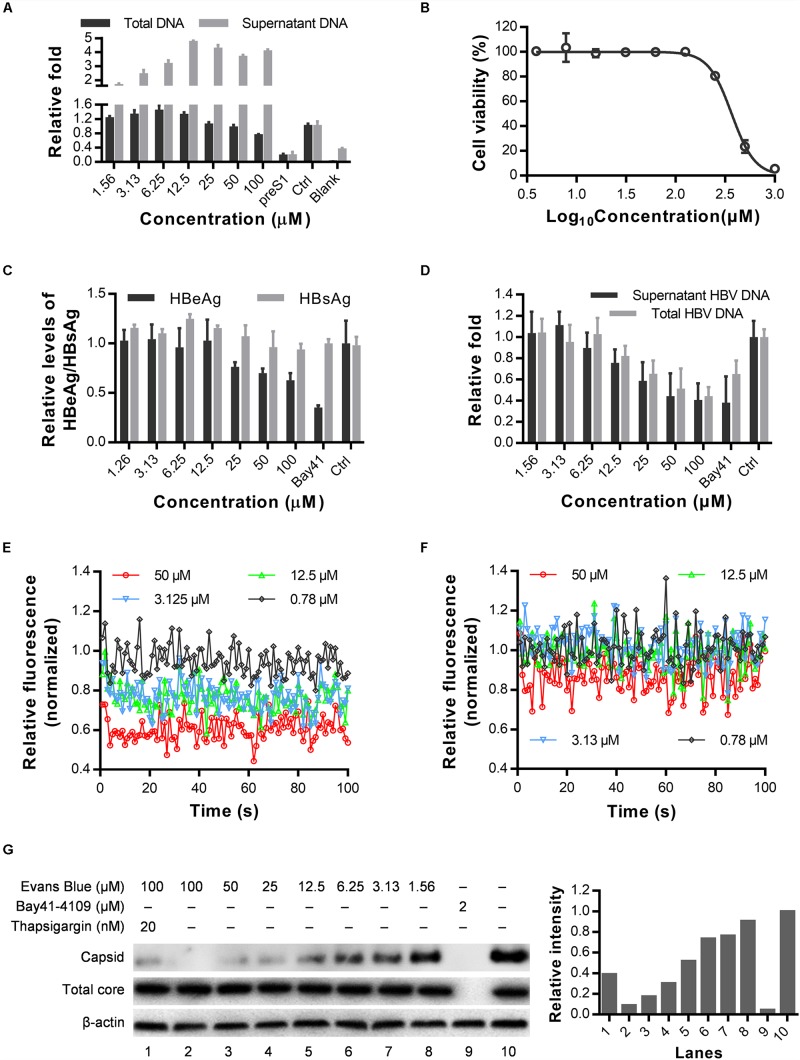
Cytosolic calcium outward induced by Evans blue is required for HBV capsid assembly. **(A)** HuhD^hNTCP^ was treated with compounds after infection for 6 days, supernatant HBV and total HBV DNAs were detected by real-time PCR. **(B–D)** Drug toxicity and antiviral effects of long-term (6 days) treatment of Evans blue on HepAD38 cell line. The antiviral effects of Evans blue treatment were determined by HBeAg, HBsAg, total DNA, and supernatant HBVs. **(E,F)** Cytosolic calcium levels in HepAD38 cells treated with Evans blue alone **(E)** or with Evans blue and 10 μM thapsigargin **(F)** were determined using a calcium dye. **(G)** HepAD38 cells plated in 6-well plates were treated with different concentration of Evans blue for 6 days, and the HBV core protein levels were detected by Western blot, β-actin was used as an internal reference. A particle gel assay followed by western blot was conducted to measure the capsid levels.

As a BK_Ca_ (large-conductance Ca^2+^-activated K^+^ channel) stimulator, Evans blue can induce Ca^2+^-dependent outward currents in sheep bladder myocytes (Fu et al., 2015). Cytosolic calcium is essential for HBV replication, core assembly, and induction of cell death (Choi et al., 2005; Xia et al., 2006; Geng et al., 2012). We, therefore, speculated that Evans blue might affect HBV capsid assembly through mediating calcium outflow. As shown in Figure 5A, we found that HBV total DNAs in HBV infected Huh7D^hNTCP^ have decreased concentration-dependently upon Evans blue treatment. However, the supernatant HBV virion numbers represented by HBV copy numbers have increased when treated with Evans blue at higher concentrations. We hypothesized that Evans blue might inhibit the re-infection of progeny virions and thereby lead to an accumulation of virions in the supernatants. Then we used HepAD38, an HBV stable-transfection cell line, to assess Evans blue’s effect on capsid assembly. The CC50 of Evans blue on HepAD38 cells was determined to be 351.8 μM (Figure 5B). Next, Evans blue was found to inhibit HBV weakly based on the supernatant HBeAg levels using Bay41-4109 for positive control. HBV copies in the supernatants and total HBV DNAs in cells, however, were reduced concentration-dependently upon Evans blue treatment (Figures 5C,D). Meanwhile, the cytosolic Ca^2+^ concentrations in HepAD38 cells treated with Evans blue and thapsigargin were measured using Fluo4 AM, a fluorescence indicator of intracellular Ca^2+^. Evans blue was found to decrease cytosolic calcium in a concentration-dependent manner (Figure 5E). Thapsigargin, which increases the cytosolic Ca^2+^ concentration, can rescue Evans blue mediated Ca^2+^ outflow and counteract its effect on HBV replication (Figure 5F). We next explored whether Evans blue affects the assembly of HBV. As expected, the reference compound, Bay41-4109, an HBV assembly inhibitor, inhibited the assembly of HBV through degraded core protein. Whereas Evans blue inhibited the assembly of HBV capsids in a concentration-dependent manner without affecting the levels of HBV core protein. Evans blue reduced the capsid level by more than 50% at 12.5 μM, and the inhibitory effect was rescued by thapsigargin treatment (Figure 5G). In sum, Evans blue reduces cytosolic Ca^2+^ level to block capsid assembly and the production of mature HBV particles.

### Evans Blue Inhibits the Infection by Drug-Resistant HBV Strains in Huh7D^hNTCP^ Cells

**FIGURE 6:**
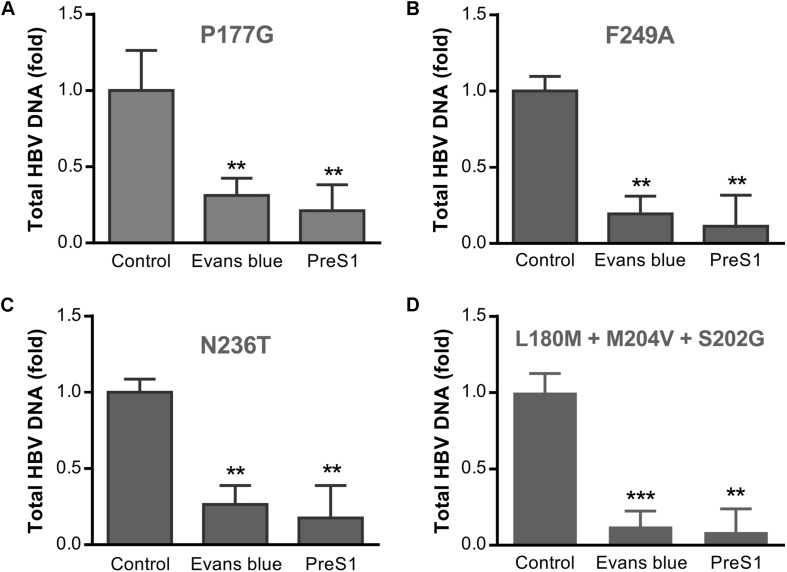
Evans blue inhibits the infection of drug-resistant strains of HBV. Huh7 cells transfected with an HBV plasmid carrying drug resistance mutation(s) as described in Section “Materials and Methods” were cultured in DMEM media for 4 days. The supernatant virions were collected and used to infect Huh7D^hNTCP^ cells at Geq = 2000. Six days after infection, total HBV DNAs were extracted for PCR analysis. Drug-resistant mutations P117G **(A)** and F249A **(B)** are tenofovir resistant, N236T **(C)** is adefovir resistant, and L180M + M204V + S202G is 3TC/ETV-dual-resistant **(D)**. Statistical significance was determined using Two tails Student’s *t*-test (^∗^*P* < 0.05, ^∗∗^*P* < 0.01, ^∗∗∗^*P* < 0.001).

Long term treatment with NAs has produced many drug-resistant variants of HBV, which may lead to treatment failure. To assess the antiviral effects of Evans blue on the infection of the common drug-resistant variants of HBV, we tested four drug-resistant variants: two tenofovir resistant variants (rtP177G and F249A), an ADV resistant variant (rtN236T), and a 3TC/ETV-dual resistant HBV variant (L180M + M204V + S202G). We transfected the four drug-resistant HBV-expressing plasmids into Huh7 cells and purified HBV particles in the supernatants. HBV infection assay was conducted with the treatment of Evans blue or preS1 as a positive control. Real-time PCR quantitation of HBV total DNA on day 6 post-infection showed that Evans blue significantly inhibited the replication of all four drug-resistant HBV variants (Figure 6), suggesting that HBV NA-resistant strains are sensitive to Evans blue treatment.

## Discussion

In this study, we identified Evans blue as a new HBV inhibitor by screening an FDA drug library using Huh7D^hNTCP^ cells. We further confirmed the antiviral effect of Evans blue using PHH. The mechanism of action study revealed that Evans blue has a dual antiviral effect against HBV infection. Specifically, Evans blue blocks the attachment of HBV to the host membrane through NTCP mediated preS1 binding, and HBV capsid assembly by affecting cytosolic calcium outflow. During the attachment step of HBV entry, Evans blue interacts directly with NTCP to affect its function but does not degrade it. During HBV capsid assembly, Evans blue induces cytosolic calcium outflow, which interferes with capsid assembly.

Evans blue has been approved for use in animals and humans and was proven safe. Both the pharmacological and toxicological profiles of Evans blue have been characterized and published. Evans blue has been widely used for detecting blood-brain barrier disruption (Hawkins and Egleton, 2006), and recently was identified as a BK_Ca_ channel stimulator. It is also widely used to study blood vessel and cellular membrane permeability, as it is non-toxic over a wide range of concentrations (Hamer et al., 2002). Evans blue can inhibit the binding or fusion of HIV type I and II to CD4^+^ T cells by interfering with gp120 function (Pal et al., 1991). Evans blue can also inhibit various DNA polymerases (Nakane et al., 1986) and protein tyrosine phosphatases (PTPases) (Shrestha et al., 2004). However, there have been no reports on Evans blue’s inhibitory effect on HBV. Here, we have found that Evans blue efficiently inhibits HBV infection in Huh7D^hNTCP^ cells and PHH and Evans blue can also prevent capsid assembly by inducing outward calcium flow.

Hepatitis B virus initiates infection of host cells by binding of the virally encoded preS1 to the host membrane protein NTCP, which is encoded by the gene SLC10A1. NTCP belongs to the solute carrier (SLC10) family, which includes seven genes, only three of which mediate sodium-dependent uptake of organic substrates across the cell membrane. These include apical sodium-dependent bile acid transporter, sodium-dependent organic anion transporter, and NTCP (Döring et al., 2012). NTCP is a nine transmembrane protein with one site (amino acids 157–165) required for binding to LHBs (Yan et al., 2013). HBV first attaches to HSPGs and then binds to NTCP through LHBs. Next, endocytosis and fusion happen through unknown mechanisms. HBV entry is essential for the initiation, spread, and maintenance of HBV infection. Drugs that block this step can effectively inhibit HBV reinfection after liver transplantation and vertical transmission. Combination therapy with NAs and entry inhibitors is a new therapeutic regimen for CHB. Myrcludex-B is a modified HBV preS1 peptide currently in a phase II study that can inhibit HBV binding at very low concentrations (Urban et al., 2014). CsA and vanitaracin A inhibit HBV binding to NTCP by disrupting the bile acid transport activity of NTCP (Nkongolo et al., 2014; Kaneko et al., 2015). Whereas two CsA derivatives, SCY450 and SCY995, have no significant effect on NTCP transporter activity (Shimura et al., 2017). EGCG and Ro41-5253 inhibit HBV infection by decreasing NTCP expression and inducing NTCP degradation (Huang et al., 2014; Tsukuda et al., 2015). Also, three FDA-approved agents, irbesartan, ezetimibe, and ritonavir, which are known as NTCP transporter inhibitors, can reduce LHBs-dependent viral infection (Lucifora et al., 2013; Blanchet et al., 2014; Wang et al., 2015). Evans blue shows significant potential to inhibit HBV infection, since the IC_5__0_ and CC_5__0_ for Huh7D^hNTCP^ are 2.1 and >1000 μM, respectively (Figure 1C), suggesting a selective index of greater than 500. We assume that the molecular mechanism of Evans blue is to bind NTCP directly, and consequently affect the binding of HBV-LHBs to NTCP. Furthermore, we found Evans blue inhibits the infection of several of the most common drug-resistant HBV strains in Huh7D^hNTCP^ cells (Figure 6), suggesting that Evans blue inhibits HBV through a different target (host factor NTCP) from that (HBV polymerase) of NAs.

It is worth noting that Donkers et al. (2017) reported that Chicago sky blue 6B, which has an alternate name: direct blue 1, can block HBV preS1 binding to NTCP and HBV infection in HepaRG cells. Since Evans blue is similar structurally to Chicago sky blue 6B, we, therefore, tested a panel of analogs that have similar structures to Evans blue or Chicago sky blue 6B for anti-HBV binding activity in Huh7D^hNTCP^ cells. Unexpectedly, all analogs except Evans blue did not show any anti-binding effects or anti-HBV effects at the maximum non-toxic concentration (50 μM) (Figure 1D and Supplementary Figure 1A). Then we increased direct blue 1 concentration to 200 μM and it showed inhibition slightly on FITC-preS1-myr binding to NTCP (Supplementary Figures 1B,C). Analogs of Evans blue and Chicago sky blue 6B at gradient concentrations from 1.9 to 50 μM were also added to Huh7D^hNTCP^ during infection to evaluate anti-HBV effects, however, none of them except Evans blue showed inhibition on HBeAg level in the supernatant at 6 dpi (Supplementary Figure 1D). These results suggest that the dual antiviral mechanism of Evans blue on HBV by targeting virus binding and capsid assembly depends highly on its own specific structure.

The secondary antiviral effect of Evans blue is the inhibition of HBV capsid assembly. The HBV core protein, a 21 kD HBV structural protein, can assemble to form the capsid with the involvement of pgRNA and other host factors, such as SRPK1, SRPK2, CDK2, and PLK1 (Liaw et al., 2010; Ludgate et al., 2012; Diab et al., 2017). Capsid assembly is a pivotal step in the late stage of the HBV life cycle, which is responsible for the formation of the mature virion and recycling of the cccDNA pool. The inhibition of HBV capsid assembly is also an anti-HBV strategy. Currently, the major HBV capsid inhibitors can be grouped into two classes. The first class, such as Bay41-4109, inhibits capsid formation by directly reducing the HBV core protein level. The second class, such as AT-61, prevents pgRNA encapsidation into nucleocapsids without affecting self-assembly of the core protein. Recently, three L-type Ca^2+^ channel inhibitors: lomerizine, cilnidipine, and nifedipine, have been identified to inhibit HBV replication. We have found another L-type Ca^2+^ channel inhibitor, oxethazaine, which inhibits HBV capsid assembly by blocking capsid self-assembly without changing the core protein level. As a BK channel stimulator, Evans blue can reduce cytosolic Ca^2+^ concentration in HepAD38 cells, thereby leading to a reduction in HBV capsid formation and capsid associated DNA levels. Twelve μM Evans blue inhibited nearly 50% of capsid formation, and the CC_50_ of Evans blue on HepAD38 cells was around 352 μM. Western blot analysis showed that Evans blue did not affect HBc protein expression. The inhibitory effect of Evans blue on capsid formation was rescued by 2 nM thapsigargin, indicating that the decrease of Ca^2+^ in the cytoplasm induced by Evans blue is the cause of capsid assembly inhibition.

Evans blue is an azo dye that has been used as an *in vivo* dye for over 50 years. Its biological toxicity is very weak (>850 mg/kg in rats) and it maintains its structural integrity without being degraded easily *in vivo*, which makes it last longer at the appropriate dose in clinical use. Evans blue has high water solubility and can be administered through different routes. Most importantly, Evans blue exhibits a dual anti-HBV effect. One is the inhibition of virus binding to host cells through the host factor NTCP, and the other is the inhibition of capsid assembly through the host factor BK channel. The barriers to the development of Evans blue-resistant HBV strains may be high since Evans blue targets host factors. However, the drugability of Evans blue needs to be improved through a chemical modification to increase its efficacy and to decrease its binding to albumin. Therefore, further evaluation of Evans blue as a potential novel anti-HBV therapy is warranted.

## Data Availability Statement

All datasets generated for this study are included in the article/Supplementary Material.

## Author Contributions

XC and YX conceived and designed the experiments and contributed to the writing of the manuscript. YX, CL, WT, and HZ performed the experiments. YX, CL, and XC analyzed the data.

## Conflict of Interest

The authors declare that the research was conducted in the absence of any commercial or financial relationships that could be construed as a potential conflict of interest.
