# Supplementary figures and images for: Evans Blue Inhibits HBV Replication Through a Dual Antiviral Mechanism by Targeting Virus Binding and Capsid Assembly
Source: Front Microbiol. 2019 Nov 14;10:2638. doi: 10.3389/fmicb.2019.02638 (PMC6868041; doi:10.3389/fmicb.2019.02638)

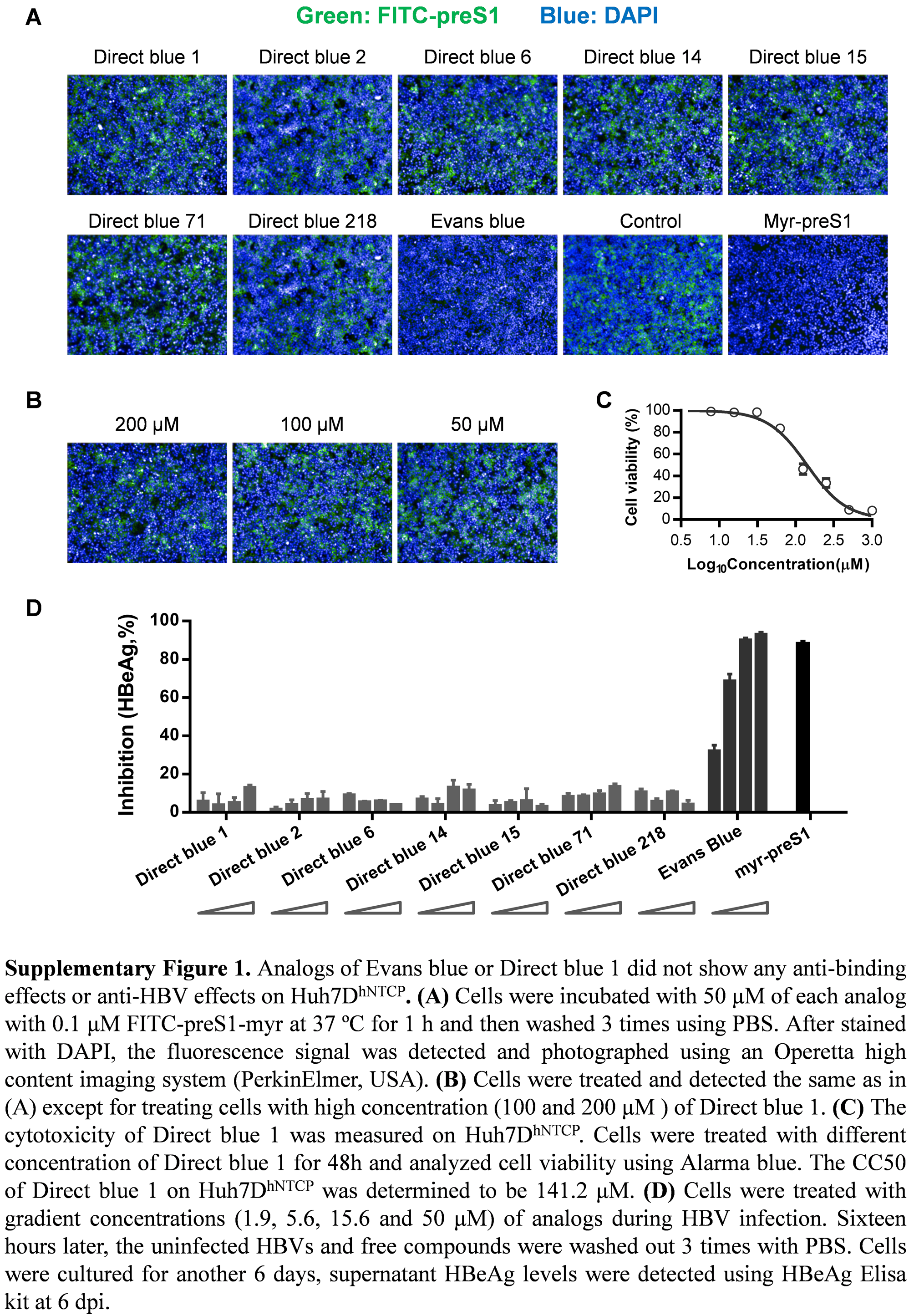

Supplement: Supplementary file 1 [file Image_1.TIF]
